# Nurturing the Next Generation: Health Challenges in Infants and Children Across Asia and Africa

**DOI:** 10.7759/cureus.42573

**Published:** 2023-07-27

**Authors:** Shreyash Agrawal, Mayank Kumar, Swarupa Chakole

**Affiliations:** 1 Department of Community Medicine, Jawaharlal Nehru Medical College, Datta Meghe Institute of Higher Education and Research, Wardha, IND

**Keywords:** under-five diarrhea, premature infants, infants, morbidity and mortality, morbidity, pre-term birth

## Abstract

Over the past 20 years, there has been a global improvement in the health of the world's population. For instance, the number of illnesses among children under five years old has been reduced by half in the last 40 years. Unfortunately, in the past decade, these positive trends have reversed in many parts of sub-Saharan Africa and some areas of South Asia. Asia and Africa carry the highest disease burden worldwide. The lack of adequately trained healthcare professionals in the public sector, as well as inequalities based on social, financial, and geographical factors, contribute to high mortality rates in Asian and African countries. Infants and children in lower-middle-income countries are particularly vulnerable to these healthcare system inequities. While the global under-five mortality rate has decreased by half in the last two decades, this progress is not observed in African and Asian countries, where the situation may even be worse in some cases. Mortality indicators, although crucial for assessing health status and making global comparisons, fail to fully capture the disease burden and healthcare utilization. Morbidity indicators, which provide insights into the prevalence of diseases, are underutilized due to limited data availability, ineffective reporting, and gaps in data storage and analysis. This article explores the morbidity data from two Asian and two African countries in an attempt to understand the most common health challenges faced by infants and children in these regions.

## Introduction and background

Diseases that can cause death often bring about substantial health complications as well. This can include conditions such as congenital anomalies, premature birth, sepsis, malnutrition, and infectious diseases in children, which all contribute to the overall burden of disease [1]. By monitoring the trends of morbidity, healthcare systems can become better prepared to address the needs of their populations.

While tracking mortality rates is important in assessing health status and making global comparisons, it does not provide a complete picture of the impact of disease and the utilization of healthcare services. Mortality indicators only capture the number of deaths caused by a disease, but they do not reflect the number of people who are living with the disease, the severity of the disease, or the impact the disease has on a person's quality of life [2].

Morbidity indicators, on the other hand, provide valuable information on the impact of diseases and their effects on individuals and populations. However, these indicators are rarely used due to a lack of data, poor reporting methods, and gaps in data storage and analysis. This makes it difficult to accurately measure the extent of disease and the need for healthcare services. To better understand the health needs of populations, it is important to use a combination of both mortality and morbidity indicators [3].

## Review

Methodology

**Figure 1:**
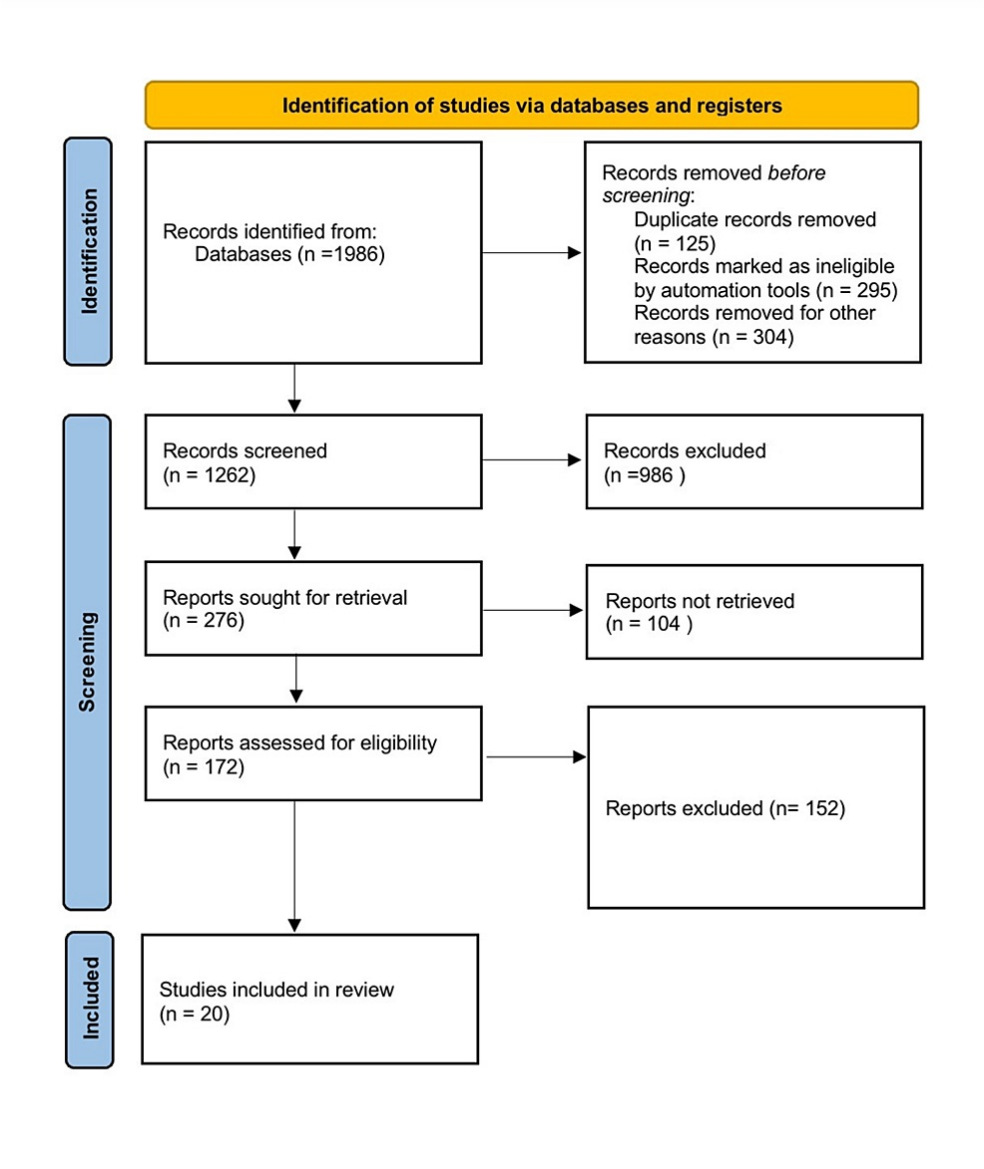
PRISMA diagram for search. PRISMA, Preferred Reporting Items for Systematic Reviews and Meta-Analyses

The terms "preterm birth," "asia-africa," "morbidity," "infants," "diarrhoea," and "malnutrition" were searched for in a database like ‘PubMed’. Only results pertaining to the English language were shown. If there was more than one published report from a similar study, the latest one was used. Only review articles that also had original data were taken into account. The PRISMA (Preferred Reporting Items for Systematic Reviews and Meta-Analyses) for the search is shown below (Figure 1).

Congenital anomalies

Congenital anomalies are a serious concern that often goes unnoticed in lower-middle-income countries (LMIC), where they can have a profound impact on an individual's life [3]. This high incidence can be attributed to a variety of factors, including a high birth rate, poor nutrition (such as neural tube defects caused by nutrient deficiencies), a lack of access to prenatal diagnosis services, environmental teratogens, and limited access to medical termination of pregnancy (MTP) services following prenatal diagnosis. Among all the countries that have been studied, Kenya has the highest burden of congenital anomalies. Despite the similarities in the patterns of congenital anomalies across these countries, there are also significant regional variations that must be taken into account. Many of these anomalies are preventable or treatable with simple interventions, such as taking folic acid supplements to prevent neural tube defects or undergoing pediatric surgery to correct arterial septal defects or ventral septal defects. However, the limited resources available in LMIC often make it difficult to address these problems effectively [4]. 

When considering the frequency of these diseases in relation to the population size, it becomes evident that they are not rare and profoundly affect the families caring for affected children. These children endure substantial morbidity throughout their lives, which hampers their development and overall well-being [5]. The significance of allocating resources and attention to this matter cannot be emphasized enough, as it impacts not just the affected individuals but also the families and communities who support them [4].

Preterm birth

Preterm birth, defined as a birth that occurs before 37 completed weeks of pregnancy or 259 days, is a major cause of neonatal death and long-term negative health outcomes. These children are more likely to experience cerebral palsy, sensory deficits, learning disabilities, and respiratory illnesses compared to those born at term [6,7]. The consequences of preterm birth extend far into adulthood, leading to substantial physical, psychological, and economic repercussions.

**Figure 2:**
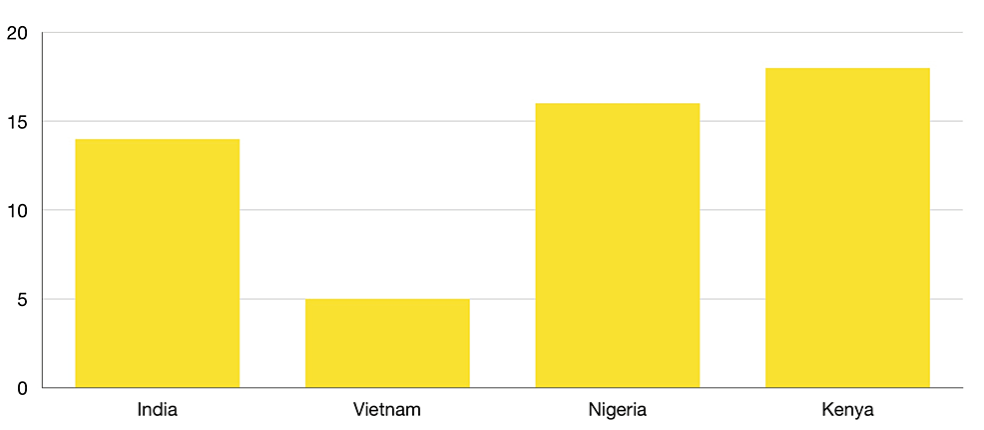
Prevalence percentage in total live births in Asian and African countries.

According to the World Health Organization (WHO), the incidence of preterm birth ranges from 5% to 18% across 184 countries, with the highest rates observed in Sub-Saharan Africa and South Asia. Figure 2 shows that African countries have a significantly higher incidence of preterm birth [8]. These high rates emphasize the importance of addressing preterm birth as a serious public health concern, as it not only affects the health of the affected children but also has wider implications for families, communities, and societies.

Pregnancy-induced hypertension, also known as preeclampsia, is a medical condition that affects many pregnant women and is one of the leading causes of preterm birth. Gestational diabetes, premature membrane rupture (PROM), premature abortion, and idiopathic causes are other common medical conditions that can lead to preterm birth. In addition, women with hypothyroidism, antepartum hemorrhage, malpresentation, and uterine overdistension are also at increased risk for preterm delivery. Mothers who have a prior history of preterm delivery are more likely to experience preterm birth again in future pregnancies. A history of abortion, intrauterine death, stillbirth, and cesarean section are also risk factors for preterm delivery.

Vaginal and urinary tract infections are common infections that are associated with adverse pregnancy outcomes, including preterm birth. These infections can cause various complications, such as inflammation and uterine contractions, which can lead to premature delivery. It is important for expectant mothers to be aware of these risk factors and to work closely with their healthcare provider to monitor their health and the health of their developing baby throughout pregnancy. Early detection and treatment of these medical conditions can help reduce the risk of preterm birth and ensure the best possible outcome for both mother and baby [9,10].

Research findings have highlighted the significant impact of anemia during pregnancy on adverse pregnancy outcomes, such as preterm birth, low birth weight, and increased maternal mortality rates [10]. The risks associated with this condition are amplified when the mother has a history of anemia before pregnancy. The combination of high neonatal and maternal mortality rates, coupled with a high fertility rate and low per capita income, presents numerous disadvantages for the population in these countries. One study suggests that providing healthcare services to pregnant mothers and ensuring skilled assistance during labor through certified healthcare activists can have a multiplier effect to improve outcomes [11]. In South Asian countries, expanding the coverage of many interventions could potentially save over a million lives but would require a cost of up to US$ 1.76 billion. Similarly, extending healthcare services to grassroots levels in sub-Saharan Africa could save up to 800 thousand lives at a cost of US$ 1.32 billion. In these regions, there are significant challenges regarding high neonatal mortality rates and preterm births. Therefore, affordable medical interventions play a critical role in improving access to healthcare services. Another study identified key factors contributing to poor neonatal outcomes in African countries, including limited facility coverage, particularly in urban areas, low family income, and inadequate public expenditure on healthcare infrastructure. Given the already unstable state of these economies, relying solely on the public sector for coverage is not a viable option. Private enterprises offering affordable solutions could create a win-win situation, considering the substantial demand for medical care. Additionally, targeted efforts by non-governmental organizations working in this sector can positively impact the current situation [12,13].

Malnutrition

Malnutrition, according to the WHO, signifies deficiencies, excesses, or imbalances in a human being's acquirement of energy and/or nutrients [12]. Malnutrition is of two types. One is "undernutrition," which includes stunting, wasting, being underweight, and micronutrient deficiencies or insufficiencies (a lack of essential vitamins and minerals). The other is overweight, obesity, and diet-related non-communicable diseases (such as heart disease, stroke, diabetes, and cancer) [12]. Child malnutrition is a significant public health problem in poor countries, which is the most important reason for child morbidity and death [14]. The nutrition of infants and young children is a significant concern to any society. Underdevelopment is the cause of severe health issues that need essential importance in children [15]. Once damage due to malnutrition sets in, in early childhood, it is challenging to alleviate the condition later on. There may be catch-up development with proper nutrition, but the time lost to disability significantly affects learning and thriving in society [16]. With urbanization in most developing countries, children are malnourished, not just undernourished, in these nations giving rise to unique challenges for the health system. The Green Revolution primarily took place in Asia because the necessary infrastructure, such as roads and seed companies, already existed, making resources readily available. However, these commodities were scarce in Africa, limiting the spread of the Green Revolution to the continent. The Green Revolution played a crucial role in reducing malnutrition among infants and children, which is a significant societal challenge. Every child deserves an opportunity to thrive and flourish [17].

Diarrhea 

Diarrhea is a significant health problem for under-five children in developing countries. Four billion cases of diarrhea were estimated to occur each year, with >90% occurring in developing countries [18]. Loose motion is stated as the expulsion of three or more soft or watery stools per day. Frequent passing of normal stools is not loose motion, nor is the passing of open, "pasty" stools by breastfed babies. Viruses are the leading causes of diarrhea in children accounting for approximately 70-90% of cases. Bacteria such as Shigella, Salmonella, Campylobacter, enterotoxigenic *E. coli*, and less regularly enter invasive *E. coli* are a causal factor in 10-20% of cases [18]. Several types of research have concluded that the betterment of potable water along with sanitation facilities leads to a decline in the peril of diarrhea. Such advancement may possibly consist of water purifiers' supply of adequate and hygienic water provided through pipe systems and proper drainage systems. Other implementable habits which can aid the reduction of diarrheal instances include regular and appropriate sanitization of hands using soaps and other means. Another prevalent cause of diarrhea is open defecation. In poorer countries of Africa and Asia, open defecation is prevalent as no proper sanitation facility is available. The fecal material defecated openly causes many diseases, including diarrhea, to run rampant. The problem aggravates especially in the rainy season when most of the cases are recorded [19]. A marked 13% to 14% reduction was seen in the cases of diarrhea when proper sanitation along with hand washing habits was instilled among the people. Bodily openings and interfaces such as mouth and hands are the primary drivers of infections among the human population. Hence the importance of washing hands regularly is highlighted by the fact that global hand washing day is celebrated all over the world on October 15. It is one of the main activities through which many diseases can be prevented from happening in the first place. Hand washing is integral to disease prevention in all parts of the world. But the major hindrance in this pathway is the inaccessibility among the least and underdeveloped countries regarding soap and other sanitary products. Impure water is yet another cause behind diarrheal occurrences. Lack of access to clean and safe potable water is still a luxury in many parts of the world. A drastic decrease of 88% in child mortalities was seen when sanitation and pure drinking water facility were made available due to diseases like diarrhea [20]. Chlorine handling of water has shown the condensed risk of diarrheal disease. Rotavirus was accountable for around 6% of cases pertaining to diarrhea and 20% of case fatalities associated with it in infants and neonates belonging to developing and underdeveloped nations. The usage of rotavirus to make virus attenuated antidote in trials in 1985 obtained a minor slump in overall diarrheal illness occurrence [20]. Similarly, inoculation against cholera displayed a notable reduction in illness and death through the oral effect of jab that was negligible as cholera is not a prominent positive disease-causing microorganism of diarrheal origin illness. Extra successful inoculation has been synthesized that has the ability to save many lives in emerging economies while reducing the complete course of treatment. Zinc supplementation proved to induce a substantial drop in occurrences of diarrhea illness contrasted to a control group [16,18]. The post-infection strategy must include the supplementation of various drugs and vitamins along with treatment. Vitamin A was found to be alleviating the situation among patients suffering from diarrhea. In addition, zinc supplementation was found to be less effective than vitamin A. Probiotics reduce the risk of diarrhea in those taking antibiotics. Insecticide spraying may lessen fly amounts and the danger of diarrhea in children in a setting where there is a cyclical variation in fly quantity during the year.

Discussion

Various socioeconomic, structural, and systemic factors come into play to affect health in LMIC. Socioeconomic factors became the most critical factor affecting the disease burden, which includes lack of employment, financial stability, and education [8]. The population cannot access the services required to mitigate the issues with a lack of resources. Most LMICs also lack the infrastructure to address the population's health needs. A majority of the health workforce is concentrated in Urban centers, and the lack of a force altogether creates a unique barrier for rural and geographically challenged people in these nations. Mitigating these issues requires a united front from the government administration. Still, a lack of commitment is observed in most nations, especially African countries, which makes the population vulnerable. Children are the most affected by such conditions; an illness in them leaves lasting effects that affect growth and quality of life [2]. These issues are not separate conditions but are interrelated in one way and depend on similar socioeconomic, cultural, structural, and systemic factors for causation and remedy. The educational status of women has a substantial impact on pregnancy and child morbidity outcomes. Low socioeconomic status is associated with women's poor lifestyle choices that lead to complications during pregnancy. Congenital anomalies were primarily found to be positively associated with alcohol and smoking. Alcohol and smoking have many side effects, which are causative factors of congenital anomalies. Inadequate water and sanitation status affect diarrhea status in children and causes infection in pregnant women, thus an associated factor in preterm birth [21]. The analysis present in disease and burden closely follows the status of major socioeconomic indicators of the countries. In comparison, Vietnam has the best socioeconomic status among the four, and the same is reflected in the burden of diseases. Vietnam saw a fast economic rise post striking economic alteration of regulations, then there were also significant progresses in healthcare conditions. The analysis highlights that African nations face socioeconomic challenges in addressing the health burden caused by these prevalent diseases. "Years lived with disability" (YLD) demonstrates the impact an illness has on quality of life before it resolves or leads to death. So if we look at YLD out of all the countries with a sizeable population, India is the worst-hit nation [22].

## Conclusions

The importance of enhancing the health of populations in Asian and African nations is evident, with a need for targeted health interventions and socioeconomic improvements. This includes measures like employment opportunities, education, and access to proper sanitation facilities. More research is required to understand the impact of morbidity and guide the development of equitable healthcare systems. Asia and Africa face a heavy burden of disease due to factors such as a shortage of trained healthcare workers in the public sector and inequalities based on social, financial, and geographical aspects. This study aimed to examine morbidity data from selected Asian and African countries, focusing on the prevalent health issues affecting infants and children. To address these challenges effectively, it is crucial to improve living standards, provide education, and ensure basic necessities while considering the unique needs of mothers and potential mothers through a gender lens. However, the utilization of morbidity indicators is often hindered by data limitations, ineffective reporting, and gaps in data storage and analysis.
